# Primordial Germ Cell Specification in Vertebrate Embryos: Phylogenetic Distribution and Conserved Molecular Features of Preformation and Induction

**DOI:** 10.3389/fcell.2021.730332

**Published:** 2021-09-16

**Authors:** Christina L. Hansen, Francisco Pelegri

**Affiliations:** Laboratory of Genetics, University of Wisconsin-Madison, Madison, WI, United States

**Keywords:** primordial germ cells (PGCs), germline specification, preformation, induction, embryonic development, germ cells

## Abstract

The differentiation of primordial germ cells (PGCs) occurs during early embryonic development and is critical for the survival and fitness of sexually reproducing species. Here, we review the two main mechanisms of PGC specification, induction, and preformation, in the context of four model vertebrate species: mouse, axolotl, *Xenopus* frogs, and zebrafish. We additionally discuss some notable molecular characteristics shared across PGC specification pathways, including the shared expression of products from three conserved germline gene families, *DAZ* (*Deleted in Azoospermia*) genes, *nanos*-related genes, and *DEAD-box RNA helicases*. Then, we summarize the current state of knowledge of the distribution of germ cell determination systems across kingdom Animalia, with particular attention to vertebrate species, but include several categories of invertebrates – ranging from the “proto-vertebrate” cephalochordates to arthropods, cnidarians, and ctenophores. We also briefly highlight ongoing investigations and potential lines of inquiry that aim to understand the evolutionary relationships between these modes of specification.

## Introduction

**FIGURE 1:**
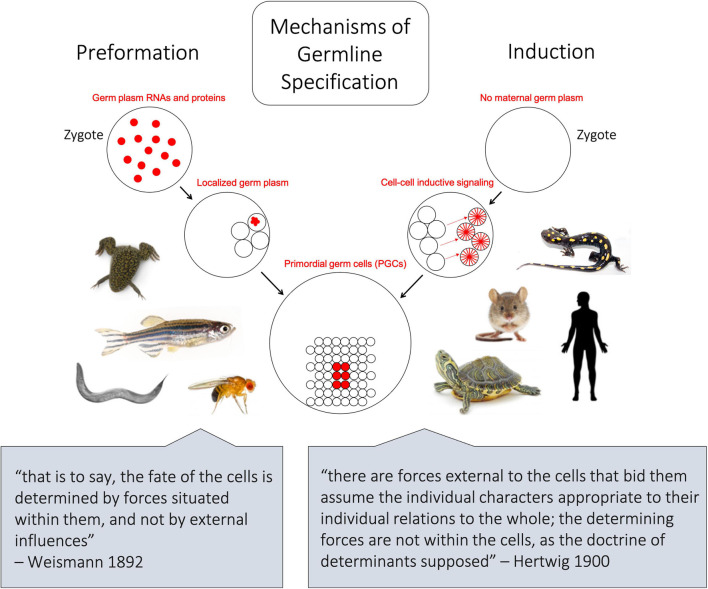
Schematic depicting the two major mechanisms of germline differentiation, preformation and induction. In preformative species, as shown on left, maternally derived germline determinants, also known as germ plasm ribonucleoparticles (GP-RNPs), are already present in the zygote and subsequently localize in a subset of cells to confer germline fate. In inductive species, as shown on right, no maternally derived germline determinants are present in the zygote, and primordial germ cells gain their identity later in embryonic development through cell–cell signaling. The lower quotes represent opposing 19th century-ideologies, from which the terms preformation and epigenesis (induction) were historically associated.

The differentiation of primordial germ cells (PGCs) from somatic cells is one of the earliest cell fate decisions in animal development. PGCs give rise to germline stem cells, which will ultimately become the progenitors of every cell in the next generation (Johnson and Alberio, 2015). Thus, the fidelity of PGC specification and development is intimately tied to the survival and fitness of sexually reproducing species. Modes of PGC differentiation fit into two main categories: induction and preformation (Extavour and Akam, 2003; Figure 1). Some vertebrate lineages, including placental mammals and urodele amphibians, use inductive cell–cell signaling interactions involving zygotic genes for germ cell determination (Johnson et al., 2011). In other vertebrates, such as teleost fish, anuran amphibians, and birds, germ cell differentiation occurs through preformation, the inheritance of maternally derived gene products necessary to confer germline fate (Houston and King, 2000; Extavour and Akam, 2003; Kloc et al., 2004). For simplicity, in this review we refer as species with either the inheritance of maternal germline determinants via preformation or the induction of PGC fate through cell-cell signaling as “preformative species” or “inductive species,” respectively. Herein, we provide an overview of the current state of knowledge of both mechanisms, including broad insights from work done in model species and how they relate to PGC specification in various non-model vertebrates. We address some of the most striking characteristics shared between these otherwise distinct specification pathways, such as conserved germline genes. Further, we detail the various germ cell determination systems among the kingdom Animalia. We will primarily discuss vertebrate species but will also include select invertebrates such as “proto-vertebrate” cephalochordates, arthropods, cnidarians, and ctenophores. Finally, we summarize active studies and potential avenues for further investigation that involve interrogating the evolutionary relationships between these means of specification.

## Principles of Preformation

The term preformation can be traced back to philosophical arguments regarding the genesis of life, wherein living organisms arose as “preformed” miniatures of their mature form (Lawrence, 2012; Figure 1). Later, in the 19th-century, evolutionary biologist August Friedrich Leopold Weismann proposed that hereditary material is transmitted by preformed determinants in germ cells, not somatic cells, as outlined in his 1892 book Das Keimplasma: eine Theorie der Vererbung (The Germ Plasm: a theory of inheritance). Although the “Keimplasma” that Weismann envisioned turned out to be a better analogy for DNA than germline determinants (Weismann, 1892; Lankenau, 2007) the concept that germ cell-specific information can be “preformed” and transferred from one generation to the next is the essence of the preformative mechanism of germline determination.

**FIGURE 2:**
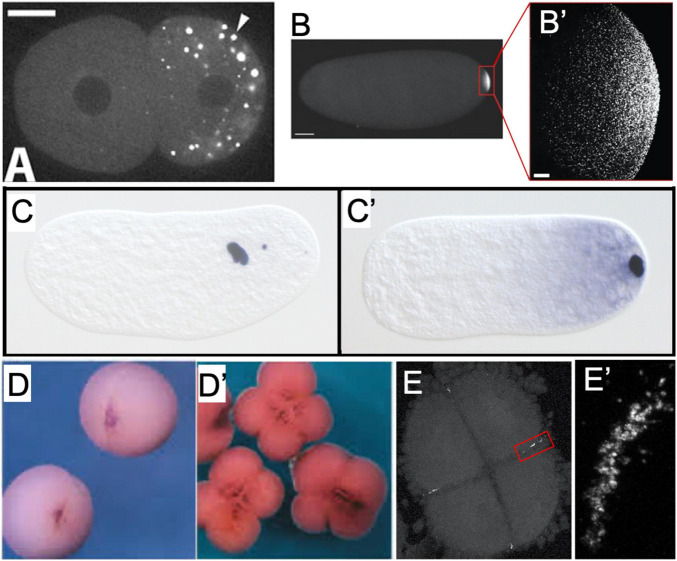
Examples of germ plasm morphology in several invertebrate and vertebrate species used for research about the preformative (maternal inheritance) mechanism of primordial germ cell specification. **(A)** PGL-1 protein-labeled P-granules in 2-cell *C. elegans* embryo. Adapted with permission from Spike et al. (2008). **(B)** Vasa protein-labeled pole plasm in a *Drosophila* embryo less than 1 h after egg laying, also magnified **(B’)** to show individual Vasa-positive germ granules that comprise the pole plasm. Adapted from Trcek et al. (2015) and available via CC-BY license. **(C)** *Nasonia vitripennis* wasp embryos in division cycle 2–3 and **(C’)** pre-syncytial blastoderm formation, labeled for the oosome component *oskar* RNA. Adapted from Lynch et al. (2011) and available via CC-BY license. **(D)** Vegetal view of germ plasm in albino *Xenopus* 2-cell and 4-cell **(D’)** embryos labeled for *dazl* RNA. Adapted with permission from Houston et al. (1998). **(E)** Zebrafish 4-cell embryo labeled for *nanos* RNA to show rod-like germ plasm masses at the cleavage furrow ends, also magnified **(E’)** to show structure of aggregated *nanos* germ plasm ribonucleoparticles.

Modern science defines preformation as a mechanism of germ cell fate driven by the presence and function of maternally inherited molecular determinants (Houston and King, 2000). Originally synthesized in oocytes, these determinants ultimately take the form of RNA and protein aggregates that segregate from the embryonic cytoplasm and confer germline fate to prospective PGCs (Figure 2). Although the terminology for these maternally inherited determinants can vary between species and developmental stage, germ plasm (from “germinal cytoplasm”) is a generally used descriptor of these determinants in vertebrate embryos.

Germ plasm was one of the earliest identified phase-separated subcellular structures (also known as membraneless organelles or biocondensates) and is characteristically electron-dense when imaged with electron microscopy (Mahowald, 1962). Phase separation, the formation of multiple distinct phases (regions of space/material with essentially uniform physical properties) from a single homogenous mixture, permits the segregation of cellular compartments from the rest of the cell without the use of a membrane and is proposed to serve critical biochemical functions by concentrating macromolecules and enabling efficient reactions, as well as protecting components from degradation (Banani et al., 2017; Uversky, 2017). While some germline-specific biocondensates, such as Balbiani bodies (large proteinaceous and organelle-rich spheres, also called mitochondrial clouds) in oocytes and perinuclear nuage in meiotic cells, are prevalent in both preformative and inductive animal species, the aggregation of maternally inherited germline determinants in early embryogenesis is specific to preformative species (Nott et al., 2015). Germ plasm components in vertebrates typically aggregate at or near embryonic cleavage furrows (Figures 2D–E’) and can be associated with cytoskeletal elements (Whitington and Dixon, 1975; Moravec and Pelegri, 2020), which promote their asymmetric inheritance into a subset of cells that will become germline progenitors.

Much of what we know about preformation in vertebrate species has been gleaned from studies involving the model developmental systems, *Danio rerio* (zebrafish) and *Xenopus laevis* (African clawed frog). However, even in these relatively well-studied species, the complete composition of germ plasm is unknown, with fewer than two dozen RNAs and protein components presently characterized in each species, and limited overlap across those species (Cuykendall and Houston, 2010; Karimi et al., 2018; Ruzicka et al., 2019). This is a mere fraction of the number of germ plasm-associated components identified in invertebrate model systems such as *Drosophila* and *C. elegans* (Harris et al., 2020; Larkin et al., 2021). It is currently unclear if vertebrates with germ plasm, as in fish and frogs, simply have fewer total components or if there are many additional gene products yet to be uncovered.

Maternally inherited germline determinants in both vertebrates and invertebrates interact with aggregation-prone, intrinsically disordered germ plasm “organizer” proteins, including Bucky ball (Buc) in zebrafish (Bontems et al., 2009), Xvelo1 in *Xenopus* (Nijjar and Woodland, 2013), and Oskar in *Drosophila* (Lehmann, 2016). Buc and Xvelo1 have limited sequence homology, with a shared N-terminal BUVE (Buc-Velo) motif containing a prion-like domain; however, neither protein appears to share any sequence homology or evolutionary origins with Oskar despite being functionally equivalent (Boke et al., 2016; Krishnakumar et al., 2018). Although beyond the scope of this review, there are several excellent sources of information about intrinsically disordered germ plasm organizing proteins, and phase separation more broadly, in germline development of many species (reviewed in Dodson and Kennedy, 2020; Mukherjee et al., 2021; So et al., 2021). Here, we present a brief overview of the mechanistic details of preformation in two well-characterized vertebrate species: zebrafish and *Xenopus* frogs (also see Aguero et al., 2017).

### Zebrafish

**FIGURE 3:**
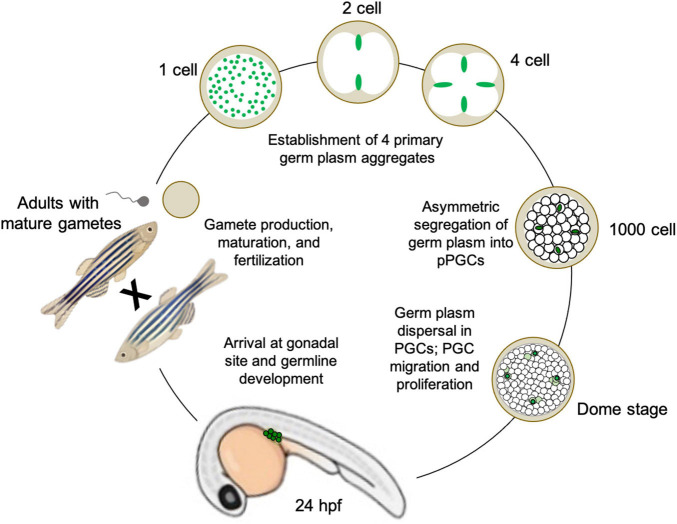
Illustration of germline determination cycle in the preformative model species *Danio rerio* (zebrafish), from maternally derived germ plasm dynamics during early embryogenesis to gamete maturation and reproduction during adulthood.

During zebrafish oogenesis, germline-specific ribonucleoparticles (RNPs) containing RNAs (such as *nanos*, *dazl*, and *vasa*; see Section “Conserved gene families critical for PGC specification across divergent species and differentiation mechanisms”) are sequestered into the Balbiani body, a phase-separated structure that is conserved among vertebrate oocytes (Figure 3; Olsen et al., 1997; Yoon et al., 1997; Braat et al., 1999; Köprunner et al., 2001; Hashimoto et al., 2004; Kosaka et al., 2007). Upon oocyte maturation and subsequent fertilization, the majority of these germline RNPs localize throughout the future blastodisc (animal pole), while a subset localizes to the yolky vegetal pole (Hashimoto et al., 2004, 2006; Howley and Ho, 2000). During the first embryonic cell cycle, cytoskeletal elements and components of the cell division apparatus mediate the movement of animal pole germline RNPs to both distal ends of the forming cleavage furrow (Yoon et al., 1997; Pelegri et al., 1999; Eno and Pelegri, 2018). Here, the RNPs are compacted into large aggregates, while the second cell cycle repeats the process of gathering RNPs to both ends of the second cleavage furrow. Simultaneously, the subset of germline RNPs that had previously been localized to the vegetal pole migrate to join the animal pole RNP aggregates at the distal furrow tips (Theusch et al., 2006). At the end of the second cell cycle, there are four large (∼50 micron length) germ plasm aggregates of RNA and protein germline determinants, which will be maintained and asymmetrically inherited throughout the subsequent 3 h of embryogenesis (Braat et al., 1999; Knaut et al., 2000; Eno and Pelegri, 2013; Figures 2E, 3).

To date, there have been 11 mRNAs and one miRNA identified as germ plasm components in zebrafish, in addition to at least five proteins. Although they are densely packed together to form the supramolecular aggregate structures, individual germ plasm RNPs are homotypic, i.e., they have only one RNA type occupying each particle, throughout the cleavage and blastula stages (Eno et al., 2019). By sphere stage, the four germ plasm aggregates are still contained within four cells (or cell clusters, reflecting occasional germ plasm fragmentation into two daughter cells rather than the more typical asymmetric segregation into one). At dome stage, approximately 4.3 h post-fertilization, the germ plasm aggregates disperse as RNPs, which have maintained their homotypic character and fill the cytoplasm of their resident cell, fated as a presumptive PGCs (Knaut et al., 2000; Eno et al., 2019). From that point on, the germline determinants segregate into both daughter cells during cell division, allowing the pool of PGCs to increase before they migrate to the genital ridge and initiate the germline developmental program, which includes further proliferation followed by meiotic commencement (Knaut et al., 2003; Raz and Reichman-Fried, 2006; Hsu et al., 2018; Figure 3).

### Xenopus

As in zebrafish, the majority of *Xenopus* RNAs and proteins destined for germ plasm localization are initially collected into the densely packed Balbiani body during oogenesis (Kosaka et al., 2007; Nijjar and Woodland, 2013). As oocyte development progresses, however, these germline determinants are concentrated primarily to the vegetal pole through multiple localization pathways [known as the early (message transport organizer or METRO), late, and intermediate/dual pathways] (Forristall et al., 1995; Kloc and Etkin, 1995; Houston, 2013). After fertilization, small germ plasm aggregates are collected through the action of surface contraction waves and microtubule associations, and ingress along vegetal cleavage furrows (Ressom and Dixon, 1988; Savage and Danilchik, 1993; Oh and Houston, 2017). Unlike the meroblastic cleavage characteristic of zebrafish embryos, where only the animal pole is cellularized and undergoes cleavage, *Xenopus* embryonic cleavage is holoblastic, taking place across the entire embryo and associated with germ plasm localization in the vegetal blastomeres (Figures 2D,D’). As in zebrafish, *Xenopus* germ plasm aggregates are asymmetrically segregated into a single daughter blastomere after each subsequent division until gastrulation, when symmetric cell division commences and the PGC pool increases to approximately 20 – 50 cells (Dziadek and Dixon, 1977; Kamimura et al., 1980). During the late tailbud stages 3 days post-fertilization, the PGCs migrate to the genital ridge where they continue to proliferate before beginning meiosis and gametogenesis (Al-Mukhtar and Webb, 1971; Kloc et al., 2004). Despite some contrasting characteristics, such as different patterns of cleavage and incomplete overlap of germ plasm components, the underlying mechanism of PGC specification in *Xenopus* and zebrafish, and indeed all known preformative animals, is the same: aggregation and subsequent asymmetric segregation of maternally derived, germline fate-promoting RNAs and proteins.

## Insights Into Induction

**FIGURE 4:**
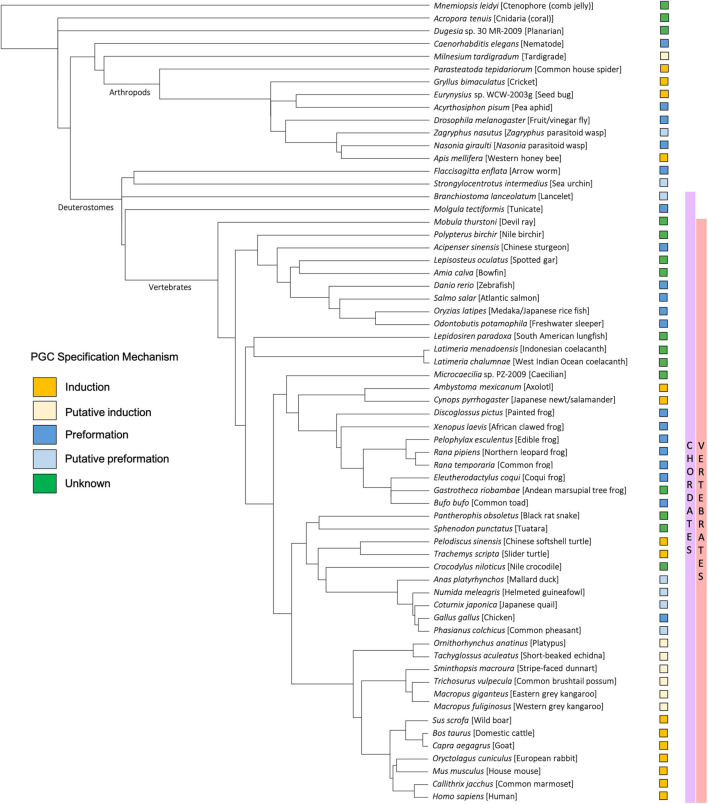
Taxonomic tree generated using the NCBI Common Tree Taxonomy Browser and Interactive Tree of Life (Letunic and Bork, 2021) to depict each species or clade referenced in this manuscript. Primordial germ cell (PGC) specification mechanism of each species is indicated according to key.

As with preformation, induction (also known as epigenesis) originated as a philosophical position held by Aristotle and others that life develops anew and is susceptible to external forces, rather than as the inevitable maturation of preformed determinants (Maienschein, 2017). In animals that employ the inductive mode of germline development, no package of cytoplasmic germline determinants is set aside in the oocyte. Instead, PGCs gain their identity later, at approximately the early gastrulation stage of embryogenesis, by receiving signals driven by zygotic gene products in neighboring tissue (Johnson et al., 2011). Thus, for induction, the key to germline fate is extracellular context rather than intracellular content (Figure 1). Much of our knowledge regarding inductive germ cell determination in vertebrates is the result of studies within mammalian systems, particularly in mice, but also in humans, pigs, and cows (Hayashi et al., 2018), and within certain urodele amphibian systems, such as the axolotl (Johnson et al., 2003a). Genes important for PGC induction in these species, including members of the bone morphogenic protein (BMP) signaling pathway (Hammerschmidt and Mullins, 2002; Lochab and Extavour, 2017) and transcription factors such as *Tfap2c* (Hoffman et al., 2007) and *Blimp1/Pdrm1* (Wilm and Solnica-Krezel, 2005), also play crucial roles in the early development of preformative species; however, in the latter, they are associated more broadly with body plan and tissue patterning rather than directing PGC fate. Induction has been proposed to be the ancestral mode of PGC specification, largely due to the presence of preformation in multiple derived lineages (presumably through convergent evolution) whereas induction is often associated with basal phylogenetic branches (Johnson et al., 2003a; Ewen-Campen et al., 2013; Figure 4). In general, the inductive mode of PGC specification requires the coordination of several zygotically driven processes in PGC precursors: repression of somatic fate, activation of germline-associated and pluripotency genes, and epigenetic reprogramming (Kumar and DeFalco, 2017).

### Mice

**FIGURE 5:**
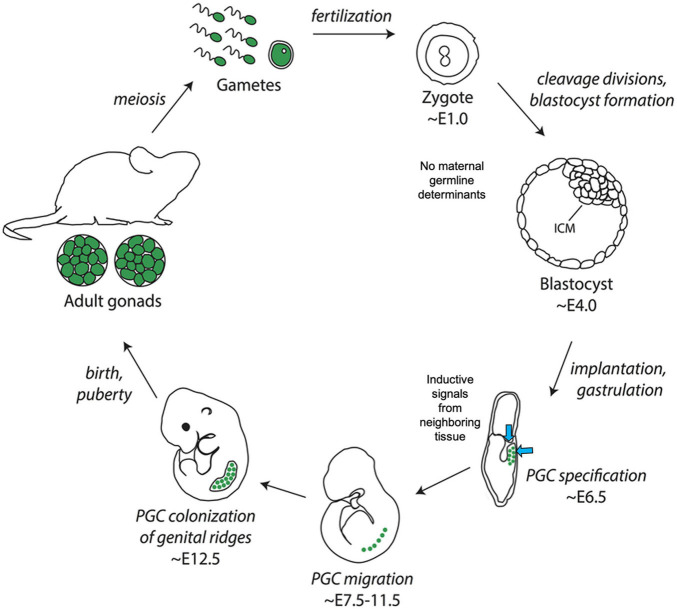
Illustration of germline determination timing in the inductive model species *Mus musculus* (mouse). Note the lack of maternally derived germ plasm in embryos and relatively late onset of primordial germ cell specification at approximately ∼E6.5. Image adapted from Richardson and Faulkner (2017) and available via license: Creative Commons Attribution-Non-Commercial 4.0 International.

In mice, expression of the transcription factors *Blimp1/Prdm1*, *Prdm14*, and *AP2-gamma/Tfap2c* is the earliest known signature of PGC specification, at approximately embryonic day 6.25 (early gastrulation) (Kojima et al., 2014; Figure 5). Expression of these transcription factors is thought to be induced by a combination of signaling events, including the secretion of bone morphogenetic protein 4 (BMP4) from nearby cells in the extra-embryonic ectoderm and the simultaneous expression of WNT3 in the posterior visceral endoderm of the epiblast (Magnúsdóttir and Surani, 2014). Together, BLIMP1/PRDM1, PRDM14, and AP2-gamma/TFAP2C promote the expression of certain germline-specific genes, such as *Nanos3*, and repress genes involved in somatic differentiation, such as the Hox family genes (Magnúsdóttir et al., 2013). By embryonic day 7.5 (late gastrulation), the complementary processes of somatic program repression and germline gene induction result in the establishment of 30–40 PGCs (Figure 5). PGCs in mice, along with humans and pigs, do not irreversibly commit to germline fate until after migration to the genital ridge, at approximately the tailbud stage, which is relatively late in development compared to germline lineage commitment in preformative species (Nicholls et al., 2019).

### Axolotl

Embryological studies of the Mexican salamander *Ambystoma mexicanum* (axolotl) dating back to the mid-20th century established that PGC fate can be induced in axolotl embryos from animal cap (primitive ectoderm) tissue (Smith, 1964; Kocher-Becker and Tiedemann, 1971; Boterenbrood and Nieuwkoop, 1973; Sutasurja and Nieuwkoop, 1974; Maufroid and Capuron, 1977; Michael, 1984), and are not readily identifiable through histological means until the establishment of the lateral mesoderm (Humphrey, 1925). Although the axolotl Dazl homolog (*Axdazl*) is expressed maternally, its RNA is not specifically localized in oocytes and remains widely distributed until after PGC migration to the developing gonad, suggesting against preformation as the mode of PGC specification (Johnson et al., 2001). However, the issue was not definitively settled until 2014, when Chatfield and colleagues confirmed that the fibroblast growth factor (FGF)- and BMP4-driven signaling pathways are responsible for PGC induction in axolotl embryos. As is the case in mice, axolotl PGC development begins with embryonic intercellular signaling (albeit involving mesodermal precursors in the ventral marginal zone as opposed to extra-embryonic ectoderm in mice) that stimulates the transcription of conserved germline-specific factors, such as *dazl* and *vasa*, and requires the simultaneous suppression of somatic fate (Chatfield et al., 2014). Similarly to other mice and other inductive vertebrates, axolotl PGCs do not appear to irreversibly commit to germline fate until a much later stage of development than in preformative animals, with germline commitment occurring after gastrulation at approximately the tailbud stage, when many somatic lineages have already been established (Chatfield et al., 2014; Nicholls et al., 2019).

## Conserved Gene Families Critical for PGC Specification Across Divergent Species and Differentiation Mechanisms

Some of the most striking characteristics shared between the two main PGC specification pathways, preformation and induction, include the prevalence of conserved germline genes (Juliano et al., 2010; Lesch and Page, 2012). Although there is also evidence of tolerance to variation, with several examples of apparent species- or genus-specific PGC genes (see section “Discussion”), here we will focus on a handful of gene families common in germline research: *DAZ* (*Deleted in Azoospermia*) genes, *nanos*-related genes, and *DEAD-box RNA helicases*. These gene families were selected based on the vast abundance of information available about them within the scientific literature and their established importance in PGC specification and reproductive processes across diverse animal species. Molecular products of these genes are also notable for their involvement in germ cell maintenance and gametogenesis, including progression through meiosis (Kotov et al., 2014; Fu et al., 2015; De Keuckelaere et al., 2018). Additionally, these genes have been shown to have sexually dimorphic functions during germ cell and gonadal development (Kraemer et al., 1999; Raz, 2000; Xu et al., 2001; Tsuda et al., 2003; Abdelhaleem, 2005; Saga, 2008; Lasko, 2013; Fu et al., 2015). This list is not exhaustive; due to space limitations, we are omitting other notable conserved PGC-related gene families, such as *PIWI/Tudor* genes and the recently characterized *GCNA* (*Germ Cell Nuclear Antigen*) gene family (Siomi et al., 2010; Carmell et al., 2016; Bhargava et al., 2020). However, the three selected gene families, *DAZ* (*Deleted in Azoospermia*) genes, *nanos*-related genes, and *DEAD-box RNA helicases*, provide an informative illustration of the deep evolutionary relationship between PGC specification pathways even amongst disparately related species.

### DAZ (Deleted in Azoospermia) Genes

**FIGURE 6:**
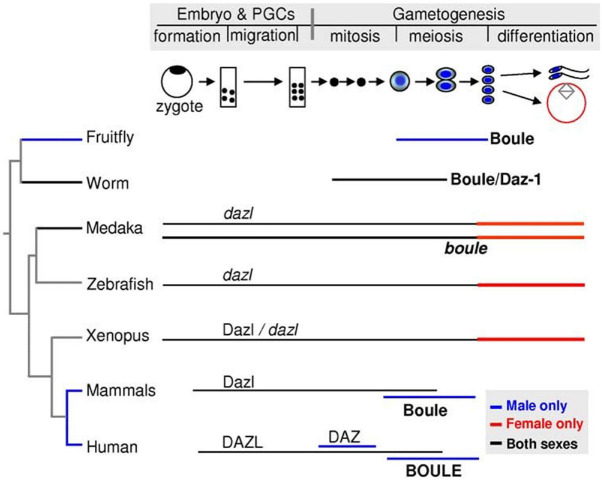
Expression summary for the DAZ family genes *Boule*, *Daz*, and *Dazl* across stages of primordial germ cell specification and gametogenesis in selected model organisms. Image adapted from Xu et al. (2009) and available via CC-BY license.

The Daz family is an ancient and broadly conserved group of genes that play a particularly prominent role in animal PGC and/or germ cell development, regardless of specification mode (Xu et al., 2001). In general, proteins encoded by DAZ family genes contain at least one conserved stretch of 24 amino acids rich in Asn, Tyr, and Gln residues, known as a DAZ repeat, and an RNA recognition motif (RRM) (Fu et al., 2015). DAZ genes are split into three branches: (1) *Boule*, the ancestral form, is conserved across metazoans; (2) *DAZ-like (Dazl)* is conserved across vertebrates; and (3) *DAZ* is only present in certain primates, including humans. Depending on the species and developmental stage, each DAZ family gene may contribute to germ cell specification in a slightly different way. In humans, mutation and/or deletion of any of the four *DAZ* genes on the Y chromosome often lead to low or absent sperm concentration and is the leading cause of male infertility (Yen et al., 1997; Fu et al., 2015). However, out of the three branches of DAZ family genes, only *Dazl* is thought to be widely necessary throughout the entirety of germ cell development, including in germline progenitors, within vertebrate species (Xu et al., 2001; Smorag et al., 2014; Fu et al., 2015; Figure 6).

Although molecular evidence related to PGC specification in non-model mammalian species is scarce, overexpression of *Dazl* in pig and goat somatic stem cells enhanced *in vitro* transdifferentiation into germline lineage and entry into meiosis (Liu et al., 2009; Park et al., 2013; Yan et al., 2015). These results, together with studies in mouse *Dazl* mutants, suggest that *Dazl* may be broadly active in these roles across most, if not all, mammalian species. *Dazl* RNA is also present in germ cell development in inductive non-mammalian vertebrate species, as evidenced by its expression in the PGCs and/or germ cells in Chinese soft-shell turtles (Xu et al., 2018) and axolotls (Johnson et al., 2001) although evidence of its functional roles during pre-meiotic stages of PGC development is lacking in these species.

*Dazl* is also a key contributor to PGC development in preformative vertebrate species. Maternally inherited *dazl* mRNA is a well-established component of germ plasm in amphibians and fish, with detailed reports of its localization in the model species *Xenopus laevis* (Houston et al., 1998) and *Danio rerio* (Maegawa et al., 1999; Theusch et al., 2006) in addition to observations in a multitude of other preformative species, such as *Pelophylax* (Rana frogs) (Marracci et al., 2011) and *Odontobutis potamophila* (dark sleeper fish) (Zhu et al., 2018). Depletion of maternal *dazl* mRNA in *Xenopus* causes reduced PGC numbers in adolescent animals (Houston and King, 2000); similarly, in medaka, knockdown of maternal Dazl protein via antibody neutralization led to reduced PGC formation (Li et al., 2016). Despite the differences between the performative and inductive mechanisms of PGC specification, these phenotypes in frogs and fish ultimately resemble the outcome of DAZL loss in mice and humans: germ cell loss and reduced fertility (Zagore et al., 2018).

### Nanos-Related Genes

As with the DAZ family, Nanos-related genes are also omnipresent in PGC development across both inductive and preformative species (De Keuckelaere et al., 2018). Initially described in *Drosophila* (Wang and Lehmann, 1991), one or more copies of germline-associated *Nanos* genes have also been identified across all investigated animal species, including invertebrates (Juliano et al., 2010), basal vertebrate species (Gribouval et al., 2018), and model vertebrates mice (Tsuda et al., 2003), axolotl (Chatfield et al., 2014), *Xenopus* (Mosquera et al., 1993; MacArthur et al., 2000), and zebrafish (Köprunner et al., 2001). Nanos RNA is a maternally inherited germline determinant and component of germ plasm in most known preformative species and localizes to PGCs via a conserved sequence in its 3′UTR (Škugor et al., 2014). Nanos RNA is regulated by other genes associated with PGC development, such as the vertebrate-specific protein Dead-end 1 (Dnd1). In zebrafish, Dnd1 recognizes a site within the nanos 3′UTR and protects nanos RNA from miR430-dependent degradation in the developing germline (Kedde et al., 2007), while Dnd1 in *Xenopus* appears to promote the translation of Nanos protein after fertilization (Aguero et al., 2017).

Nanos proteins are required for PGC survival during zygotic stages of embryogenesis and maintenance of oocyte production during adulthood in both preformative and inductive species, such as Drosophila, mice, and zebrafish (Draper et al., 2007). As a group, Nanos proteins contain a conserved C-terminal (CCHC)2 zinc finger motif which enables them to bind a variety of RNAs and proteins (Curtis et al., 1997; De Keuckelaere et al., 2018). Pumilio, an RNA-binding protein and founding member of the PUF family, is one well-characterized example of a Nanos binding partner (Zamore et al., 1997; Nakahata et al., 2001; Jaruzelska et al., 2003). Nanos and Pumilio proteins form a translation repressor complex, best described in the preformative species Drosophila but also conserved in the inductive germline development of humans, that promotes germ cell maintenance by preventing somatic gene expression and apoptosis in PGCs (Forbes and Lehmann, 1998; Jaruzelska et al., 2003; Weidmann et al., 2016).

### DEAD-Box RNA Helicases

Perhaps the most well-known molecular marker of the germline is the DEAD-box RNA helicase Vasa/DDX4 (Gustafson and Wessel, 2010; Hickford et al., 2011). Vasa/DDX4, as with all DEAD-box proteins, contains multiple conserved motifs spanning two main functional domains that enable ATPase and RNA helicase activity. Phase-separated perinuclear Vasa/DDX4 protein granules are a tell-tale marker of germ cells in both preformative and inductive animal species (Gustafson and Wessel, 2010). Although these Vasa/DDX4-positive perinuclear granules generally form after the specification of PGCs, with functions more closely associated with meiosis, they may also support PGC migration and proliferation (Raz, 2000; Kistler et al., 2018).

Maternally inherited *vasa/ddx4* mRNA is highly expressed as a germ plasm component in zebrafish embryos (Yoon et al., 1997) along with model invertebrate preformative species such as *Drosophila* and *C. elegans*. In contrast, in the Japanese rice fish *Oryzias latipes* (medaka), neither transcripts nor protein encoded by the *vasa* homolog *olvas* exhibit germ plasm localization in cleavage stage embryos. This finding was so unexpected that it initially called into question whether or not medaka specify their PGCs via preformation (Shinomiya et al., 2000; Kurokawa et al., 2006). However, electron micrographs of 4-cell stage medaka embryos revealed electron-dense structures highly characteristic of germ plasm appearance in cleavage furrows, and perturbations of BMP2 signaling did not impact PGC number – two pieces of evidence that strongly suggested that medaka do indeed undergo preformation instead of induction, but just happened to lack germ plasm-associated *vasa/ddx4* (Herpin et al., 2007). The *Xenopus vasa*/*ddx4* homolog, Xvlg1, also lacks distinctive germ plasm localization, with expression in both somatic and germ cells, but mRNA from a different DEAD-box RNA helicase, DEADSouth/*ddx25*, is localized to *Xenopus* germ plasm and required for PGC development (MacArthur et al., 2000; Yamaguchi et al., 2013). In mammals, such as mice and humans, Vasa/DDX4 protein is expressed in migrating PGCs and exhibits sexually dimorphic functions in germ cells during gametogenesis (Fujiwara et al., 1994; Castrillon et al., 2000; Tanaka et al., 2000; Noce et al., 2001). Vasa/DDX4 and another DEAD-box RNA helicase, GRTH/DDX25, are associated with granular nuage-like structures in mammalian spermatogenic cells and required for spermatogenesis and male fertility (Tanaka et al., 2000; Tsai-Morris et al., 2010; Tutak and Rozwadowska, 2020).

## Distribution of Germ Cell Determining Systems Across Phylogeny

Although much of what is known about germ cell determination in vertebrate species has been garnered from studies involving model developmental systems, there is also an ever-increasing amount of information gained from less commonly used vertebrate models. Here, we first summarize what is known (and still unknown) about the distribution of germ cell determining systems amongst diverse species in each of the five major vertebrate classes. We also briefly address the distribution of germ cell determining systems in invertebrate lineages, including *Drosophila* and *C. elegans*, in addition to a broad survey of other invertebrate species, from tunicates to tardigrades.

### Amphibians

**FIGURE 7:**
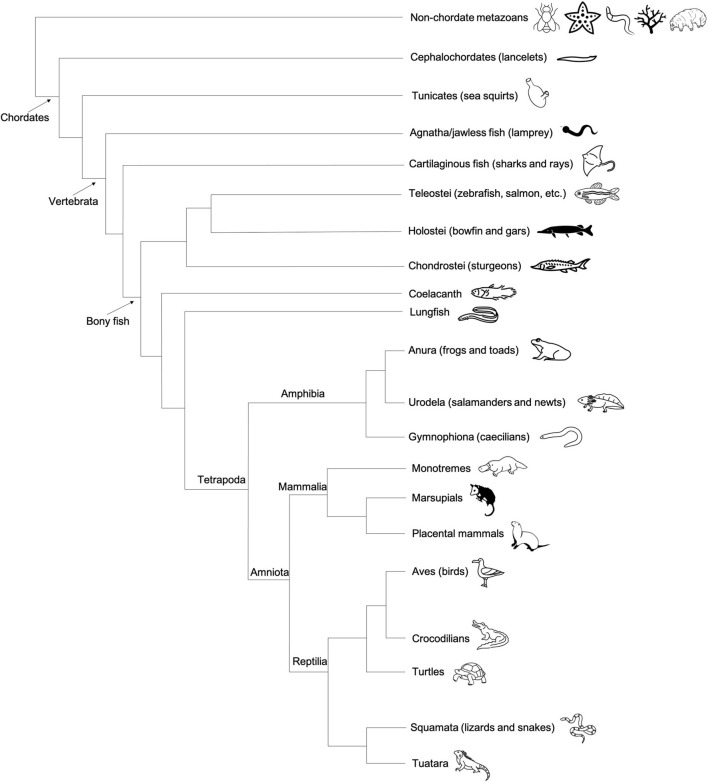
Cladogram depicting the general evolutionary relationships between major vertebrate classes, invertebrate chordates (tunicates), and non-chordate metazoans. Animal graphics adapted from the Noun Project (https://thenounproject.com/) and available via CC-BY license.

Amphibians are categorized into three phylogenetic classes: Anura (frogs and toads), Urodela (salamanders and newts), and Gymnophiona (caecilians) (Figure 7). As with *Xenopus* frogs (see section “*Xenopus*”), many other anuran amphibians, such as *Rana temporaria* (European common frog), *R. pipiens* (Northern leopard frog), *Bufo bufo* (Common toad), *Discoglossus pictus* (Mediterranean painted frog), and *Eleutherodactylus coqui* (Common coquí) exhibit the molecular markers characteristic of the preformation system of germline specification (Bounoure et al., 1954; Smith, 1966; Czolowska, 1969; Elinson et al., 2011). In all of these species, the dense granules characteristic of maternally inherited germ plasm are promptly apparent following fertilization at the vegetal pole, and germ plasm masses are asymmetrically inherited by a subset of embryonic cells, presumptive PGCs, which give rise to the eventual germline (Smith, 1966; Czolowska, 1969).

Unlike anuran species, which have been found to use the preformation mechanism for PGC specification, studies in axolotl salamanders suggest urodele amphibians use induction to specify PGCs (see also above, section “Axolotl”). Further supporting the assumption that urodele amphibians as a group do not possess germ plasm, a lack of maternal *Dazl* RNA localization during early embryogenesis had previously been reported in at least one other urodele species, *Cynops pyrrhogaster* (Japanese newt) (Tamori et al., 2004). The germline specification system used by the snake-like, limbless caecilians, members of the amphibian class *Gymnophiona*, is currently unknown. Caecilians are considered a basal lineage relative to both urodeles and anurans, so knowledge of their PGC determination mechanism could be particularly useful from an evolutionary perspective.

### Avian and Non-avian Reptiles

In modern phylogenetic contexts, reptiles and birds are grouped into the monophyletic class Reptilia (Figure 7). As with the class Amphibia, the bulk of embryological and gene expression evidence suggests that a subset of Reptilia, including avian species, specify germ cells via preformation, whereas at least some non-avian reptiles use the inductive mechanism (Figure 4).

In 2000, the identification and subsequent localization studies of chicken Vasa homolog (CVH) (Tsunekawa et al., 2000) in chicken embryos provided strong evidence of an avian species using preformation to specify germline cells. Reminiscent of germ plasm aggregate localization in the cleavage furrows of zebrafish and *Xenopus* embryos, maternally inherited CVH protein accumulates within the first cleavage furrow of chicken embryos and is asymmetrically inherited into a small subset of cells (∼6–8 out of 300) as embryonic development progresses. These cells, the presumptive PGCs, proliferate and subsequently circulate through the vascular system and migrate to the germinal ridges where they initiate gonadogenesis (Tsunekawa et al., 2000; Nakamura et al., 2013; Kim and Han, 2018).

Another PGC marker, *DAZL* RNA, was found to be specifically localized in chicken oocytes and embryonic cleavage furrows, further supporting the conclusion that chicken specify PGCs via maternal inheritance of germline determinants, rather than induction (Lee et al., 2016). Research involving germ cell development in other avian species, such as quail, duck, pheasant, and guinea fowl have primarily been in the context of isolation and *in vitro* production of PGCs in hopes of generating long-term PGC culture systems and transgenic progeny, rather than elucidating the early mechanistic details of germ plasm aggregation and maintenance (Reynaud, 1981; Ono et al., 1996; Kang et al., 2008; Wernery et al., 2010; van de Lavoir et al., 2012; Chen et al., 2019). Analysis of PGC specification *in vitro*, integrated with emerging studies in developing embryos, should provide valuable insight into avian germline specification.

In contrast to avian reptiles, research in turtle (*Trachemys scripta*) oocytes demonstrated that *dazl* and *vasa* are not localized, unlike the specific localization pattern during oogenesis of preformative animals. Additionally, identification of turtle PGCs originating in the posterior crescent (reptilian embryonic equivalent of mammalian early posterior primitive streak), as opposed to during the cleavage stages, provided suggestive evidence of the inductive system of germ cell specification (Bachvarova et al., 2009b). This was further supported by similar findings of posterior localization of PGCs in a lizard (*Lacerta vivipara*), while other species of lizards exhibited PGC localization in both the posterior and anterior crescents. PGC localization in snake (*Vipera aspis*) embryos more closely resemble their progression in birds, leading some to propose that snakes may also specify germline fate via the preformation mode instead of induction (Bachvarova et al., 2009a; Bertocchini and Chuva de Sousa Lopes, 2016). The PGC specification system in crocodilians, which are more closely related to birds than other reptiles such as lizards, is unknown.

### Fish

Fish, the most abundant vertebrate group, can be split into three major classes: Agnatha (jawless fish, e.g., lamprey), Chondrichthyes (cartilaginous fish, e.g., sharks), and Osteichthyes (bony fish, e.g., salmon) (Figure 7). Much of what we know about germ cell development in fish is the result of studies of a handful of species within the teleost clade of bony fish, which represents an estimated 96% of the approximately 30,000 known living fish species (Volff, 2005). In addition to extensive experimental evidence of maternally derived germ plasm in zebrafish, similar signatures of localized maternal determinants have been identified in at least a dozen other teleost fish spanning hundreds of millions of years of evolutionary history, including *Salmo salar sebago* (Atlantic salmon), *Acipenser sinensis* (Chinese sturgeon), *Oryzias latipes* (Japanese rice fish/medaka) (Saito et al., 2004; Herpin et al., 2007; Škugor et al., 2016). In addition to teleosts, there are two other infraclasses of bony fish: Holostei (bowfin and gars) and Chondrostei (sturgeons and birchirs) (Figure 7). The identification of localized germ plasm RNA in multiple sturgeon species suggests that Chondrostei use preformation for germ cell specification (Saito et al., 2014). In holosteans, which are more closely related to teleosts than chondrosteans, the distribution of germ cell specific RNAs has not yet been described. In some basal fish species, such as lungfish and coelacanths, evidence of germ plasm localization in oocytes and early embryos is also lacking and requires further investigation (Johnson et al., 2003a). The PGC specification method used by non-bony fish (jawless or cartilaginous) is also currently unknown.

### Mammals

Placental mammals, or eutherians, is the largest group of extant mammalian species, including humans, rodents, and others that serve as models for various biological processes. As with mice, induction of PGC specification in rats (Kobayashi et al., 2020) involves the expression of signaling molecules from embryonic tissues, including the extra-embryonic ectoderm. Non-rodent placental mammals, such as humans, pigs, and rabbits, do not form extra-embryonic ectoderm tissue during embryogenesis, yet still specify their germ cells via induction. Human PGC specification *in vivo* has historically been more challenging to address than in mice, primarily due to the ethical and logistical concerns inherent to studying a prenatal process, thus, much that is known has been achieved through research involving induced pluripotent stem cells (Kobayashi and Surani, 2018). Through these studies, it is now known that several genes in the PGC specification pathway are shared by both mice and humans, such as *BMP*, *WNT*, *PRDM14*, although the molecular targets and roles of these genes are not always conserved between species (Sybirna et al., 2020). Zygotically expressed *Dazl* in the gonads of mice, humans, and pigs seems to be required for germline commitment and maintenance, suggesting that this role might be conserved across most, if not all, placental mammals (Nicholls et al., 2019).

As the vast majority of mammalian germ cell determination studies have been done in placental mammals, there is reasonable interest in uncovering the mechanism(s) used by monotremes and marsupials. In particular, monotremes, which lay eggs, diverge significantly with their reproductive strategy from other mammalian species. However, egg laying as a reproductive trait does not necessarily correlate with one PGC specification system over the other, since, as mentioned above, there are examples of egg laying species in both preformative and inductive categories. Despite being biologically interesting, resolving the PGC specification method in monotremes may not be feasible without the development of organoids/embryoids due to the critically endangered status of the only two living monotreme species, platypus and echidnas.

Marsupials, which as therian mammals are more closely related to placentals than the monotremes, pose a more realistic opportunity for PGC research. Intriguingly, transmission electron micrographs of four-cell stage embryos from the marsupials *Sminthopsis macroura* (Stripe-faced dunnart) and *Trichosurus vulpecula* (Common brushtail possum) revealed “nucleolus-like” aggregates of granular electron-dense material in the cytoplasm noted by the authors as bearing resemblance to germ plasm or nuage, albeit not localized to any specific cellular structure (Frankenberg and Selwood, 1998; Kress and Selwood, 2003). However, to our knowledge, there is no molecular evidence that germ plasm markers are localized to these electron-dense aggregates, nor evidence that these structures are retained in future PGCs. As with all other animal species investigated, the broadly conserved germline-specific RNA helicase DDX4/Vasa RNA and/or protein has been identified in several marsupial species, including tammar wallaby and opossum, and in the platypus (a monotreme). Oddly, *ddx4* mRNA is expressed at the 16 – 32 cell stages of tammar wallaby embryonic development but not at any other prenatal stage, and immunohistochemistry of PGCs within fetal gonads did not detect DDX4 protein. However, DDX4 protein is present during all stages of tammar wallaby postnatal oogenesis, and both *ddx4* mRNA and protein are highly expressed in the adult testes of tammar wallabies, in addition to platypuses and echidnas (Hickford et al., 2011). Ultimately, beyond these expression-based studies, not much is known about germline determination in neither marsupials nor monotremes, leaving much room for future investigation and potential insights into the evolution of PGC specification systems.

### Invertebrates

Although this review is written with a focus on vertebrates, we would be amiss to not briefly address the immense diversity of germ cell developmental programs in invertebrates, particularly in non-model species (for a thorough review of germline determination in model invertebrates, see Strome and Lehmann, 2007).

#### Arthropods

The largest clade of invertebrates, the Arthropoda phylum, encompasses the model system *Drosophila*, along with other insects, arachnids, myriapods, and crustaceans. Relative to other invertebrate phyla, a remarkable number of arthropod species have had their PGC determination mode characterized. Examples of both preformative species (such as *Drosophila melanogaster*) and inductive species (*Gryllus* crickets) have been observed within Arthropoda, and members of this phylum have been used extensively for systematic investigations into the evolutionary history of PGC specification methods (Ewen-Campen et al., 2013; Donoughe et al., 2014; Nakamura and Extavour, 2016). In addition to *Drosophila*, examples of arthropods with preformation established as their germline determining system include *Acyrthosiphon pisum* (pea aphid) (Chang et al., 2006) and *Nasonia vitripennis* (parasitic wasp) (Quan et al., 2019). In these species, maternally inherited germ plasm (also called pole plasm) components asymmetrically localize to the posterior of developing embryos, either as numerous small granules such as in *Drosophila* (Figures 2B,B’) and *A. pisum*, or as a single large spherical mass (oosome) in *N. vitripenni* (Figures 2C,C’; Quan et al., 2019). Subsequently, they are segregated into the germline lineage. Ablation of these asymmetrically localized determinants impairs fertility in all three species.

Embryos of another parasitic wasp species, *Pimpla turionellae*, also possess all the characteristics typical of a preformative organism, with localized maternally inherited granules that segregate to the germline (Bronskill, 2011). However, ablation of the germ granules did not prevent germ cells from being specified and resulted in normal fertility in the adult organism (Achtelig and Krause, 1971). The molecular pathway underlying this apparent compensatory mechanism is unclear, but a similar phenomenon has also been observed in non-arthropod invertebrates such as sea urchins (Yajima and Wessel, 2011) and *C. elegans* (Gallo et al., 2010). More analysis is needed to assess whether this may hint at the (current and/or ancestral) co-existence of dual PGC specification mechanisms within the same species.

Induction is the prevalent germline determination mechanism in the majority of other studied arthropod species, including *Apis mellifera* (Western honey bee), *Gyllus bimaculatus* (field cricket), *Oncopeltus fasciatus* (milkweed bug), and *Parasteatoda tepidariorum* (American house spider). Embryological and molecular studies using field crickets demonstrated that BMP and Blimp-1 signaling are key contributors to PGC specification in these insects, as is the case in mice (Lawson et al., 1999; Donoughe et al., 2014; Nakamura and Extavour, 2016). The use of induction, rather than preformation, in some of these basal arthropod species, such as *G. bimaculatus*, is often cited as evidence for induction being an ancestral PGC specification mechanism (Ewen-Campen et al., 2013; Donoughe et al., 2014; Nakamura and Extavour, 2016), although there are still many unexplored avenues for insight into the evolutionary history of this process.

#### Non-arthropod Invertebrates

Outside of *Arthropoda*, the nematode worm *Caenorhabditis elegans* represents the vast majority of detailed PGC specification research (reviewed in Wang and Seydoux, 2013). In *C. elegans*, PGCs are specified very early in embryogenesis through the preformative method, at least partially driven by the inheritance of maternally inherited germline determinants called P-granules. The P-granules, which consist of several RNA-binding proteins and RNAs, localize to a single blastomere which gives rise to the germline lineage (Figure 2A). Unlike the case in *Drosophila* (Nüsslein-Volhard et al., 1987; Santos and Lehmann, 2004), disrupted localization of P-granules does not cause sterile adults (Gallo et al., 2010). On the other hand, simultaneous knockdown of four RNA-binding proteins (PGL-1, PGL-3, GLH-1, and GLH-4) that mediate P-granule assembly can cause germ cells to erroneously exhibit somatic characteristics (Updike et al., 2014). Beyond *C. elegans*, some aspects of PGC specification and development have been reported in various non-arthropod invertebrates but a comprehensive understanding of how these systems are distributed is still lacking.

Members of Echinodermata, a sister Deuterostome taxon to Chordata, include various species of aquatic animals such as sea urchins and sea stars. To date, germ plasm has not been definitively identified in any echinoderm species, but the sMic (small micromere) lineage in cleavage-stage sea urchin embryos has been shown to accumulate maternal germline determinants Vasa and Nanos (Juliano et al., 2006; Yajima and Wessel, 2011). However, all other studied echinoderms lack the sMic cell lineage entirely (Wessel et al., 2014), and the distribution of preformation or induction across Echinodermata remains to be determined.

Outside of Deuterostoma, Chaetognaths (arrow worms, marine predatory worms), which are hermaphroditic, exhibit strong molecular evidence of preformation; in the embryos of multiple chaetognath species, a large, Vasa protein-containing germ plasm mass localizes to the first cleavage furrow and is subsequently asymmetrically segregated to the developing germline (Carré et al., 2002). Unlike Chaetognaths, tardigrades, which are phylogenetically situated between nematodes and arthropods, are thought to use induction because of apparent determination of PGCs through cell-cell signaling interactions just before gastrulation (Hejnol and Schnabel, 2005). Although the mode(s) of specification is currently unknown for species within the basal lineages ctenophora and cnidaria, genes involved in PGC development across species, such as *Vasa*, have been successfully used as molecular markers of germ cells in the cnidarian coral species *Acropora tenuis* (Tan et al., 2020).

#### Invertebrate Chordates

In addition to vertebrates, there are two extant invertebrate lineages of chordate animals: Cephalochordata and Tunicata. Tunicates are considered the closest evolutionary relatives of vertebrates, followed by the cephalochordates. There is evidence of preformation in certain cephalochordates, also known as amphioxus or lancelets, in the form of specific localization of conserved germline determinants *nanos*, *vasa*, and *tudor7* RNAs during early embryogenesis (Wu et al., 2011; Zhang et al., 2013). Tunicates are also thought to be preformative animals largely due to the identification of putative germ plasm in the species *Ciona intestinalis* (Fujimura and Takamura, 2000; Takamura et al., 2002). As with other preformative species, *C. intestinalis* germ plasm components, including Vasa protein, localize asymmetrically within the developing embryo, first to the posterior/vegetal pole of the fertilized egg and eventually to a subset of blastomeres that will give rise to the rest of the germline (Shirae-Kurabayashi et al., 2006).

### Potential Malleability of PGC Determination Systems: Considerations and Consequences Across Phylogenetic Space

Previous work has highlighted that, despite the widespread presence of the preformative PGC specification mechanisms in multiple independent lineages within vertebrates, basal branches within those same lineages exhibit an inductive mode of PGC specification; for example, turtles within the avian/reptilian lineage, urodele salamanders within Amphibia, and potentially sarcopterygians (coelacanths and lungfish) within the fish lineage use the inductive mode of specification (Johnson et al., 2003b; Johnson and Alberio, 2015). This has led to the suggestion that, at least within vertebrates, induction is the ancestral system of PGC specification, and that preformation has arisen repeatedly through apparent convergent evolution (Extavour, 2007; Johnson et al., 2011). Such convergent evolution would be presumably driven by a strong selective advantage of preformative systems as an effective means of PGC specification, perhaps in the context of rapid cell cleavage cycle. Additionally, there may be shared embryonic features that facilitate such convergent evolution of germ plasm inheritance. Although inherited as distributed particles, germ plasm components aggregate at the furrows in fish, amphibians, and birds, and this conserved feature could be facilitated by the co-option by a germ plasm particles aggregation system of already-existing mechanisms that establish the cellular furrow during cell division (Moravec and Pelegri, 2020). In this case, aggregation of germ plasm particles may rely on mechanisms similar to those acting in furrow induction (Glotzer, 2004; D’Avino et al., 2005; Theusch et al., 2006; Pollard, 2010; Nair et al., 2013; Eno et al., 2018) such as the convergence of signals at the growing ends of astral microtubules from both sides of the bipolar spindle. Thus, underlying cellular mechanisms may provide a favorable cellular landscape for the emergence, through convergent evolution, of similar germ plasm recruitment mechanisms.

In later stages of development in preformative vertebrates and invertebrates, many germ plasm RNAs are found at the nuclear envelope. This perinuclear accumulation of germline determinants, long recognized as associated with germ cells and initially termed “nuage,” is shared between preformative and inductive species (Gao and Arkov, 2013; Kulkarni and Extavour, 2017). Such conservation suggests that the nuclear envelope localization of germ cell-specific particles is a key feature required for the specification of the PGC state, possibly through local changes that can influence nuclear gene expression (Updike et al., 2011; D’Orazio et al., 2021). Presumably maternally inherited germ plasm facilitates PGC specification by providing ready-to-go factors to initiate this process, which constitutes a selective advantage. In support of this hypothesis, in preformative species nuclear envelope-associated germ cell granules appear soon after the dissociation of maternally derived germ plasm and are thus likely formed through the redistribution of products from the germ plasm mass itself (Yoon et al., 1997; Braat et al., 1999; Knaut et al., 2000; Eno et al., 2019). Under this scenario maternally inherited germ plasm components may facilitate establishing a PGC state that is subsequently maintained through zygotic gene function (D’Orazio et al., 2021). The germ plasm as a structure could thus be regarded as an “innovation” to jump-start PGC specification, in the same way that other evolutionary innovations have been proposed to facilitate early somatic development. As a notable example, the maternal gene *bicoid* is thought to be an innovation in the dipteran lineage that facilitates the formation of anterior structures in the long germ band embryonic development (involving the simultaneous determination of all embryonic segments) characteristic of this lineage (Akam, 1987; Driever and Nüsslein-Volhard, 1988; Barker et al., 2005). Thus, in this scenario, preformative mechanisms of PGC determination may have evolved to enhance the effectiveness of the germ line-soma distinction in early developmental stages. In this sense germ cell specification and its malleability through evolution may parallel morphological and gene expression divergence in somatic cells according to the “hourglass” model of morphological diversification, in which early developmental processes and zygotic gene expression show wide divergence but which nevertheless lead to a conserved “phylotypic” body plan (Duboule, 1994; Hazkani-Covo et al., 2005; Raff, 2006; Cruickshank and Wade, 2008; Heyn et al., 2014; Cutter et al., 2019; Ewe et al., 2020). These divergent early morphological include variable patterns of cell specification that need to be coordinated with patterns of aggregation of germ plasm components.

Previous work has also highlighted that acquisition of germ plasm within a lineage is associated with increased morphological diversification, as reflected by the greater number of branches in vertebrate lineages with preformative PGC mechanisms when compared to basal lineages with inductive PGC mechanisms (Johnson et al., 2003b, 2011; Crother et al., 2007). These has led to the proposal that the presence of germ plasm may decrease restraint to evolutionary changes in the (somatic) body plan compared to inductive mechanisms (Evans et al., 2014; Johnson and Alberio, 2015). In inductive systems, PGC specification depends on somatic structures and potentially even shared signaling factors also required for somatic tissues. Factors of the BMP family, for example, are required for PGC specification in various animals such as mice (Lawson et al., 1999), axolotls (Chatfield et al., 2014), crickets (Donoughe et al., 2014; Nakamura and Extavour, 2016), and colonial tunicates (Kawamura and Sunanaga, 2011), but also have a well-known role in the specification of somatic cell types and the basic embryonic body plan (Tiedemann et al., 2001; Hammerschmidt and Mullins, 2002; Mizutani and Bier, 2008; Lochab and Extavour, 2017; Zinski et al., 2018). In this scenario, changes in the patterning of early somatic structures may adversely affect the PGC specification in inductive species, leading to infertility, hence generating an evolutionary constraint. On the other hand, the ability of germ plasm to induce germ cells in a cell-autonomous manner, according to the inheritance of aggregates of maternally inherited particles, allows animal embryos to carry out germ cell specification independent of somatic development, bypassing this constraint (Crother et al., 2007; Evans et al., 2014; Johnson and Alberio, 2015). However, certain aspects of this hypothesis have been disputed by studies in invertebrate species that demonstrated a lack of evidence for an association between preformation and increased morphological or sequence-based diversification (Whittle and Extavour, 2016, 2017).

An alternative scenario to the hypothesis that germ plasm arose independently in various vertebrate lineages, and which would still be consistent with the observed distribution of PGC specification modes across species, would be the presence of an ancestral “hybrid” mechanism with aspects of both preformation and induction systems. Redundant mechanisms are common for the specification of somatic tissues during embryonic development and are thought to provide developmental robustness (Frankel et al., 2010; Félix and Barkoulas, 2015; Yevick et al., 2019), which can provide selective advantage. Similarly, robustness through pathway redundancy could contribute to assuring germ cell specification. Indeed some animal species, such as *C. elegans*, appear to have redundant mechanisms of germline determination (Gallo et al., 2010; Strome and Updike, 2015), providing precedent for species that may rely on dual mechanisms for PGC specification. Alternatively, rather than maintaining redundant mechanisms within the same lineage, germ plasm specification modes may be fluid, changing through evolutionary time from preformative to inductive and vice versa. Such redundancy and/or fluidity would be particularly prevalent in early developmental processes, which as stated above are known to change rapidly and may be subject to changes in the underlying genetic network while maintaining a constant output (in this case, PGC specification) through a process conceptually similar to “developmental system drift” (True and Haag, 2001; Ewe et al., 2020). In this scenario, the ancestral branch of the vertebrate clade has the potential to use either mechanism, with some lineages (birds, anurans, and most fish) becoming solely dependent on germ plasm inheritance and others (mammals, urodeles, and some reptiles), having lost that potential. The greater morphological divergence of preformative lineage could be linked to a selective advantage associated with lineages that have an inductive mechanism, with basal lineages, those undergoing the least morphological change, retaining an inductive mechanism. Importantly, independent lineages with inductive mechanisms suggest that *losing* a preformation mechanism may confer a selectable advantage, perhaps by freeing cellular mechanisms that now become available to other cellular processes, as previously proposed (Cooper et al., 2014; Huang and Mackem, 2014; Lopez-Rios et al., 2014).

Of note, the contrasting hypotheses regarding the evolutionary history of PGC specification mechanisms provide different interpretations for the inductive mechanism observed in placental mammalian lineages. In one case, placental mammalian PGC specification represents an ancestral state that has not acquired the preformative method; in another, it represents a derived state that has lost germ plasm as a mechanism for germline determination. Mammalian development may pose unique circumstances for germ cell determination, such as a relatively slow cell division rate, and the presence of extraembryonic structures may allow mammals to escape potential developmental constraint in the absence of germ plasm. Certainly, greater depth in our understanding of PGC specification broadly, and germ plasm across lineages specifically, will be required to distinguish between various evolutionary scenarios of PGC specification.

## Conclusion

### Looking to the Future: Finding the Way With Non-model Species

As it is likely apparent in the sections above, there is a significant imbalance of information known regarding PGC specification in model vs. “non-model” species. Recent technological advances with particular relevance to developmental genetics, such as the availability of CRISPR-Cas9 mutagenesis, genomic and transcriptomic data from an increased breadth of species, and improved *in vitro* PGC and/or “embryoid” culturing present new opportunities for previously untenable PGC research pursuits that could improve our understanding of both the function and evolution of PGC-specific genes.

Investigation of germ cell determination in closely related lineages can provide complementary information to what is currently known from studies in distantly related model systems, and may provide a greater understanding of how germline specification may be malleable in the context of development. Practically, this could be accomplished by careful morphological and molecular analysis of PGC development in species closely related to established model organisms. These efforts are already underway in several invertebrate systems, including several *Drosophila* species (Mahowald, 1977), echinoderms (multiple sea stars, sea cucumbers, and urchins) (Fresques et al., 2016) and with vertebrates such as the *Danio* and *Devario* genera within the Danionin fish subfamily (Hansen et al., in press), which could be further extended to establish other “model genera.”

Another potential avenue for further insight into the evolutionary history of germ cell differentiation systems would be characterization of currently unknown germline determination systems in species that represent potential transition points between other developmental or evolutionary categories. For example, members of the egg-brooding “marsupial frog” genus *Gastrotheca* are anurans and would therefore be predicted to use preformation for germline specification. However, some aspects of the cleavage stages of the *Gastrotheca* species *G. riobambae* (Andean marsupial tree frog) bear more resemblance to urodeles and even mammalian embryos than to anurans, exhibiting a remarkably slow first cleavage (∼12 h in *G. riobambae* vs. 1.5 h *in Xenopus*), and lack of an obvious mid-blastula transition (Elinson and del Pino, 2012). Other candidates that could be prioritized for investigation into PGC determination systems include the Tuatara (*Sphenodon punctatus* – the sole extant member of the reptilian order Rhynchocephalia), egg-laying mammals, such as the platypus and echidnas, due to their dramatically divergent reproductive strategies from other mammalian species, and cartilaginous fishes, such as sharks and rays, which are understudied in relation to bony fishes. Each of these proposed candidates represent potentially enlightening “transition” points in evolutionary history; clades of animal life that are often overlooked when compared to their relatives in more commonly studied lineages.

### Molecular Paths for Inquiry Into the Evolutionary History of Germline Specification

It has been proposed that preformation and induction represent ends of a spectrum of germline determination mechanisms, rather than exclusionary options (Seervai and Wessel, 2013). The ubiquity of certain gene families, such as *Daz*, *Nanos*, and *Vasa/DDX4*, seems to hint at deeply rooted similarities. Accordingly, in addition to identifying the PGC determination system of less commonly studied species, molecular characterization of the key players and their functions across a wide range of species will also be important goals in any attempt to better understand the evolutionary history of PGC specification.

Of course, in the journey of scientific inquiry, insights can often originate from a study of contrasts rather than similarities. The idea that genes involved in reproduction evolve rapidly in relation to other types of genes has been suggested by several groups, and accordingly, we can see variation between species, particularly within animals that use the preformation method of PGC specification. Some gene products involved in the preformative method of germline development are examples of specialized duplicates of genes well known for other physiological processes; for example, an apparently teleost-specific carbonic anhydrase (*ca15b*) is a component of zebrafish germ plasm (Wang et al., 2013; Hartwig et al., 2014). Other PGC-related genes appear to be species-specific and defy simple characterization. For example, *Germes*, a gene whose RNA and protein products are critical components of germ plasm in *Xenopus laevis*, does not have a known gene homolog in any currently sequenced non-*Xenopus* species (Berekelya et al., 2003). Careful examination of these exceptional molecular players may lead to a more comprehensive understanding of what precisely is necessary for successful formation and maintenance of PGCs, and what aspects of this process are tolerant to adaptation.

### Concluding Remarks

Primordial germ cells specification as a topic of scientific investigation is rife with potential for discovery. Here, we aimed to summarize decades of foundational work in this field, including embryological and molecular descriptions of preformation and induction as distinct mechanisms of PGC specification. The observation that across animal species, regardless of PGC determination mechanism, PGCs at later stages of development share common features, such as the presence of nuclear envelope-associated germ granules, suggests that preformation and/or induction are alternative means for the very early embryo to initiate a common germ cell pathway. Accordingly, we discuss germline gene families shared by both preformative and inductive species. We also examine the distribution of PGC determination systems across diverse animal lineages, which provides further insights into how such systems may change over evolutionary time while still maintaining the ability to initiate the germline specification program. Indeed, many of the key unresolved questions remaining in the PGC specification field concern the evolutionary history of preformation and inductive mechanisms and are topics of active debate within the research community (Swartz and Wessel, 2015; Whittle and Extavour, 2017; Krishnakumar and Dosch, 2018; Tan and Tee, 2019); for example: (1) What is the underlying reason for the conserved feature of perinuclear germ granules that may help specify the PGC gene expression program? This feature likely reflects a key aspect of germ cell specification that links biophysical features of germ cell ribonucleoparticles, overall cellular structure involving the cytoplasmic-nuclear interphase, and gene expression programs; (2) How do germ plasm components, individually and/or collectively, help initiate PGC specification, if this is indeed the case? Here, it will be important to understand mechanisms of germ plasm dispersal into the cytoplasm, and precisely how maternally inherited germ plasm RNAs, prior to and during dispersal, facilitate the activation of the germ cell gene expression program, possibly at least in part through translational regulation cascades; (3) What factors have contributed to the repeated, independent evolution of germ plasm, or alternatively, to shifting its existence and function across lineages? Germ plasm malleability across phylogenetic space may involve biophysical properties of germ plasm as adaptable biocondensates, which provides potential both at the level of reorganization of the biocondensate itself and with its interaction and response to a dynamic cytoskeleton; (4) What is the precise evolutionary history of PGC determination across phylogenetic space? Further knowledge of genomic and expression data across various model and non-model organisms, coupled to computational phylogenetic analysis of evolutionary processes will provide insight in this important question; (5) How do mechanisms of PGC determination shift through evolutionary time? This question will integrate modes of inheritance across phylogeny with embryonic and other developmental innovations, additionally in the context of the potential and constraints conferred by dynamic properties of germ granules/biocondensates. In the years to come, we anticipate and look forward to new insights into those subjects, particularly when considering the essential goal common to all PGC specification systems: development of the germline and, ultimately, reproduction.

## Author Contributions

CH wrote the manuscript and designed/compiled the figures. FP provided the critical feedback and edits. Both authors contributed to the conceptualization of this work.

## Conflict of Interest

The authors declare that the research was conducted in the absence of any commercial or financial relationships that could be construed as a potential conflict of interest.

## Publisher’s Note

All claims expressed in this article are solely those of the authors and do not necessarily represent those of their affiliated organizations, or those of the publisher, the editors and the reviewers. Any product that may be evaluated in this article, or claim that may be made by its manufacturer, is not guaranteed or endorsed by the publisher.
